# Evolutionary Optimization of Ensemble Learning to Determine Sentiment Polarity in an Unbalanced Multiclass Corpus

**DOI:** 10.3390/e22091020

**Published:** 2020-09-12

**Authors:** Consuelo V. García-Mendoza, Omar J. Gambino, Miguel G. Villarreal-Cervantes, Hiram Calvo

**Affiliations:** 1 Escuela Superior de Cómputo, Instituto Politécnico Nacional, Mexico City 07738, Mexico; cvgarcia@ipn.mx (C.V.G.-M.); jjuarezg@ipn.mx (O.J.G.); 2 Centro de Innovación y Desarrollo Tecnológico en Cómputo, Instituto Politécnico Nacional, Mexico City 07700, Mexico; mvillarrealc@ipn.mx; 3 Centro de Investigación en Computación, Instituto Politécnico Nacional, Mexico City 07738, Mexico

**Keywords:** sentiment polarity, ensemble learning, unbalanced classes, evolutionary optimization, Twitter sentiment analysis

## Abstract

Sentiment polarity classification in social media is a very important task, as it enables gathering trends on particular subjects given a set of opinions. Currently, a great advance has been made by using deep learning techniques, such as word embeddings, recurrent neural networks, and encoders, such as BERT. Unfortunately, these techniques require large amounts of data, which, in some cases, is not available. In order to model this situation, challenges, such as the Spanish TASS organized by the Spanish Society for Natural Language Processing (SEPLN), have been proposed, which pose particular difficulties: First, an unwieldy balance in the training and the test set, being this latter more than eight times the size of the training set. Another difficulty is the marked unbalance in the distribution of classes, which is also different between both sets. Finally, there are four different labels, which create the need to adapt current classifications methods for multiclass handling. Traditional machine learning methods, such as Naïve Bayes, Logistic Regression, and Support Vector Machines, achieve modest performance in these conditions, but used as an ensemble it is possible to attain competitive execution. Several strategies to build classifier ensembles have been proposed; this paper proposes estimating an optimal weighting scheme using a Differential Evolution algorithm focused on dealing with particular issues that multiclass classification and unbalanced corpora pose. The ensemble with the proposed optimized weighting scheme is able to improve the classification results on the full test set of the TASS challenge (General corpus), achieving state of the art performance when compared with other works on this task, which make no use of NLP techniques.

## 1. Introduction

Sentiment polarity refers to the opinion people have about an entity (e.g., film, service, news, etc.). Several machine learning methods have been used to automatically determine polarity of text published on Internet [1,2,3,4]. In general, polarity is automatically determined in various domains using different approaches, for example, in health prediction [5,6,7,8] or transportation [9].

The task of sentiment polarity task can be tackled as a supervised classification problem, where classes correspond to the polarity expressed in opinions (v. gr. positive or negative); classifiers are trained on tagged examples and they generate a model that relates features to the corresponding tag. Some classifiers are able to learn better particular features, while other classifiers that fail on particular cases perform well on others. Ensemble learning uses a set of classifiers to combine their predictions in different ways. [10] showed that an ensemble of classifiers is more accurate than its individual members if each of these members has an error rate less than 0.5, and they generate different errors when classifying new instances—i.e., members are accurate and diverse. A way to combine their predictions is to apply a voting strategy than can give the same weight to each classifier (hard voting) or different weights (soft voting). There are two determining factors concerning a voting ensemble that have been studied, the set of classifiers to be combined [11] and the weight assigned to each classifier [12]. This work focuses on the second problem.

Our main goal is to find the best weights assigned to each classifier in ensemble learning while using a soft voting scheme to improve sentiment polarity classification in a multiclass, unbalanced corpus. Particularly, we focus on the Spanish TASS task [13] organized by the Spanish Society for Natural Language Processing (SEPLN) (www.sepln.org). There are several challenges to tackle in this task:

- The fact that polarities are specified in four labels: positive, negative, neutral, and none. Thus, this task has to be modeled as a multiclass problem.
- The corpus we experiment with, possesses several difficulties: it is designed to have a small training subset (approximately 10%), while the test set is around 90%.
- Classes are not uniformly represented, and their distribution varies in the two subsets.

Hence, in this work, the weighting scheme for Twitter sentiment polarity in an unbalanced corpus with four possible polarity values (positive, negative, neutral, and none) is addressed through an optimization approach. This approach involves the formulation of an optimization problem, where its solution is based on the use of the differential evolution algorithm.

Despite that several works [14,15] have proposed different strategies for calculating the weighting scheme of an ensemble, the corpora used for their experiments are balanced, so that, to our knowledge, the effects of applying a weighting scheme on an unbalanced corpus have not been explored. In addition, classifiers of previous works are designed to only learn two possible outputs (positive or negative). Adjusting the weighting scheme for multiclass classification, unlike binary classification, could be more challenging when considering the number of possible combinations in the solutions. Therefore, this paper proposes a solution to optimize the weighting scheme of an ensemble to tackle a multiclassification problem with unbalanced classes.

The rest of this paper is structured, as follows: Section 2 gives details of current methods dealing with this problem. Section 3 describes some preliminaries, such as details on the selected task, classifiers, and a formal definition of ensemble learning; Section 4 presents the main proposal of this work—the evolutionary optimization of the weighted ensemble classification. In Section 5, the experiments and results are presented, and finally in Section 6 our conclusions are drawn.

## 2. Related Work

The problem of learning from unbalanced datasets has been addressed in early works, such as [16]. With the aim of improving the performance of SVMs in the imbalanced dataset context, the authors integrate over-sampling and under-sampling to balance the data and propose the ensemble of SVM (EnSVM) model in order to integrate the classification results of weak classifiers constructed individually on the processed data, and develop a genetic algorithm-based model called EnSVM+ to improve the performance of classification through classifier selection. Inspired by this work, we aimed to propose an ensemble, but focused both on linguistic features and multiclass problems.

Regarding linguistic features, ref. [17] describes a linguistic analysis framework, in which a number of similarity or dissimilarity features are extracted for each entailment pair in a data set and various classifier methods are evaluated based on the instance data that were derived from the extracted features. They compare and contrast the performance of single and ensemble based learning algorithms of datasets from the RTE1 to RTE5 challenges. They show that only one heterogeneous ensemble approach demonstrated a slight improvement over the technique of Naïve Bayes and none of the homogeneous methods were more accurate than Naïve Bayes. Nevertheless, finding an optimal combination of classifiers is still an important issue.

Over the past few years, the use of evolutionary computing techniques in classification tasks has increased because these techniques help finding an approximate solution closer to the global solution, while retaining, at the same time, independence to particular characteristics of the optimization problem, such as discontinuities, nonlinearities, the need of discrete design variables, etc. Additionally, evolutionary computing techniques are flexible in the sense that they allow merging diverse strategies in order to improve the exploration and exploitation capabilities of the algorithm in the evolutionary process. In [18], the multi-objective version of Binary Bat Algorithm with local search strategies employing social learning concepts in designing random walks is used on three widely-used micro array cancer datasets to explore significant bio-markers. A bio-inspired hierarchical model for analyzing musical timbre is presented in [19]; the model extracts three profiles for timbre: time-averaged spectrum, global temporal envelope, and instantaneous roughness. Different weight assignment for each features in ensemble learning-based classification has been applied in [20].

Related to text classification, the Arabic Text Classification system (ATC-FA) is proposed in [21]; this system combines the algorithm of Support Vector Machines (SVM) with an intelligent Feature Selection method (FS) based on the Firefly Algorithm (FA). Genetic programming has been used in [22] to generate alternative term-weighting schemes (TWSs) in text classification, allowing to improve the performance of current schemes in text classification by combining TWSs, terms (TRs), and term-document (TDRs) with a predefined set of operators.

In [23], a hybrid ensemble pruning scheme that is based on clustering and randomized search for text sentiment classification is proposed. A consensus clustering scheme is presented to deal with the instability of clustering results that consists of self-organizing map algorithm (SOM), expectation maximization (EM), and K-means++ (KM++). The classifiers of the ensemble are initially clustered into groups according to their predictive characteristics. Subsequently, two classifiers from each cluster are selected as candidate classifiers based on their pairwise diversity. The search space of candidate classifiers is explored by the elitist Pareto-based multi-objective evolutionary algorithm for diversity reinforcement (ENORA).

In [24], a model is introduced in order to predict whether a tweet contains a location or not and show that location prediction is a useful pre-processing step for location extraction. To evaluate the model, the Ritter dataset and MSM2013 dataset were used. To train the model, they tried different machine learning algorithms: the Naive Baiyes (NB), Support Vector Machine (SMO), and Random Forest (RF) using 10-folds cross validation. To optimize accuracy and true positives, the thresholds were varied (0.05, 0.20, 0.50, 0.75) for NB and RF, and for SMO was varied epsilon (0.05, 0.20, 0.50, 0.75). The conclusion was that RF and NB are the best machine learning solutions for this problem they perform better than SMO.

Usually, sets of classifiers are more accurate than the individual classifiers that integrate them when any of their individual members has an error rate of less than 0.5, and, in general, individual members have different errors when classifying new examples—that is, they are precise and diverse [10]. In recent years, deep learning methods have achieved high performance for several tasks; however, there are several problems for which a traditional machine learning approach is able to obtain state of the art results, given that an appropriate ensemble is constructed [15,25,26,27,28].

In this sense, different schemes have been tried to combine the predictions of the base classifiers that form the ensemble classification. Particularly, for the soft weighting scheme, there has been two main approaches: the use of meta-heuristic algorithms proposed by [14] and the estimation of a weighting scheme based on the probabilities of classifiers and their accuracy, as described by [15].

In [14], the use of meta-heuristic algorithms in the weighting of ensemble learning improves classification’s performance. Onan et al. proposed including a weighted ensemble learning for the analysis of the polarity opinion (positive and negative) based on differential evolution. Ensemble learning incorporates the following classifiers: Bayesian Logistic Regression (BLR), NB, Linear Discriminant Analysis (LDA), LR, and SVM. The allocation of the appropriate weighting values to classifier outputs is established as an optimization problem where precision and recall are the objective functions. Their proposal improves the accuracy of the base classifiers and other classic methods of ensemble learning.

In [15], the polarity of opinion is determined in two classes (positive and negative) of tweets of the *Stanford Sentiment140* English corpus, proposing a combination scheme of the ensemble learning of the weights for the base classifiers NB, RandomForest (RF), SVM, and LR. The proposal considers the weighting of the accuracies of each base classifier along with their probabilities of predict a negative or positive class to calculate prediction scores. According to these scores, the authors determine the polarity of the training data. If negative and positive scores are equal, the cosine similarity is calculated with other tweets in test data and the most similar tweet prediction is chosen. With ensemble learning, the accuracy of the base classifier with better precision (SVM) is improved by 0.2%.

A multiobjective optimization-based weighted voting scheme was presented in [29]. Zhang et al. [30] propose adjusting the weight values of each base classifier by using the DE algorithm. Onan et al. [14] present a static classifier selection involving majority voting error and forward search; and, Ankit and Seleena [15] consider the weighting of the accuracies of each base classifier along with their probabilities of predict a negative or positive class to calculate prediction scores. It is important to recall that the corpora used for all these experiments are balanced (Except for *First GOP debate twitter sentiment* dataset used in [15]). Additionally, classifiers of previous works are designed to only learn two possible outputs (positive or negative). This is why this paper proposes estimating an optimal weighting scheme using a Differential Evolution algorithm focused in dealing with particular issues that multiclass classification and unbalanced corpora pose.

## 3. Preliminaries

This section outlines the selected task (Section 3.1); briefly describes it as a multiclassification task (Section 3.2); gives details on selected classifiers (Section 3.3); and, formally defines the problem of ensemble classification that will be used throughout the rest of this work (Section 3.4).

### 3.1. The Twitter Sentiment Polarity Task

In this research, the Spanish TASS corpus that was organized by the Spanish Society for Natural Language Processing (SEPLN) was used. This corpus contains 68,017 tweets divided into two sets: an *E* training set with 7219 tweets and a *Z* test set with 60,798, with polarity frequencies indicated in Table 1. As can be seen, this is a strongly unbalanced corpus, additional to the particularity that the test *Z* set is more than eight times greater than the training set *E*.

This work aims to train with the *E* set to automatically classify the *Z* set of TASS in the polarities of opinion: Positive (P), Negative (N), None (NONE), or Neutral (NEU), hence the need of multiclass classification. This is detailed in next section.

### 3.2. Multiclass Classification

Classification problems can be categorized according to the number of different values that classes can have. In binary classification, there are only two mutually exclusive classes; for instance, the spam detection task has two possible outputs: spam or valid email [31]. When the classification problem has more than two possible class values, it is considered to be a multiclass problem. An example of multiclass classification is to determine whether an opinion is positive, negative, or neutral [32]. Well-known classifiers, like Decision Trees and Neural Networks, can handle multiclass problems natively, but binary classifiers can be adapted to support multiclass classification. One of the most used strategies to transform a multiclass problem to a binary problem is One vs One (OVO) [33]. This strategy divides the original data set into two-class subsets, learning a different model for each new subset. Consider the following dataset to better explain this strategy:

Classes={P,N,NEU,NONE}

$$Instances=\{I_{1},I_{2},I_{3},I_{4},I_{5},I_{6},I_{7},I_{8},I_{9},I_{10}\}$$

$$Dataset=\{\left\{I_{1},P\right\},\left\{I_{2},N\right\},\left\{I_{3},P\right\},\left\{I_{4},NEU\right\},\left\{I_{5},NONE\right\},\;\left\{I_{6},N\right\},\left\{I_{7},NEU\right\},\left\{I_{8},P\right\},\left\{I_{9},NEU\right\},\left\{I_{10},N\right\}\}$$

The OVO strategy creates a data subset for each possible combination of pair of classes. For an *m* class problem, OVO creates $\frac{m(m-1)}{2}$ data sets and each data set is used to train a binary classifier that can distinguish between different pairs of classes [34]. For the dataset above, OVO creates the following data sets:

$$Dataset_{P-N}=\left\{\left\{I_{1},P\right\},\left\{I_{2},N\right\},\left\{I_{3},P\right\},\left\{I_{6},N\right\},\left\{I_{8},P\right\},\left\{I_{10},N\right\}\right\}$$

$$Dataset_{P-NEU}=\left\{\left\{I_{1},P\right\},\left\{I_{3},P\right\},\left\{I_{4},NEU\right\},\left\{I_{7},NEU\right\},\left\{I_{8},P\right\},\left\{I_{9},NEU\right\}\right\}$$

$$Dataset_{P-NONE}=\left\{\left\{I_{1},P\right\},\left\{I_{3},P\right\},\left\{I_{5},NONE\right\},\left\{I_{8},P\right\}\right\}$$

$$Dataset_{N-NEU}=\left\{\left\{I_{2},N\right\},\left\{I_{4},NEU\right\},\left\{I_{6},N\right\},\left\{I_{7},NEU\right\},\left\{I_{9},NEU\right\},\left\{I_{10},N\right\}\right\}$$

$$Dataset_{N-NONE}=\left\{\left\{I_{2},N\right\},\left\{I_{5},NONE\right\},\left\{I_{6},N\right\},\left\{I_{10},N\right\}\right\}$$

$$Dataset_{NEU-NONE}=\left\{\left\{I_{4},NEU\right\},\left\{I_{5},NONE\right\},\left\{I_{7},NEU\right\},\left\{I_{9},NEU\right\}\right\}$$

New instances can be predicted based on majority voting.

### 3.3. Classifiers

Three classifiers were selected relying on previous results that have shown good performance [32] to create the ensemble. Because Logistic Regression and Support Vector Machines are binary classifiers, it was necessary to use the multiclass transformation strategy explained in Section 3.2. The classifying methods (referred as classifiers from now on) and their parameters are described next:

- Multinomial Naïve Bayes (NB). This is a native multiclass classification algorithm. It is based on Bayes’s theorem. It is called *naïve* because of the assumption of class conditional independence, but, in spite of this, good results are obtained with this algorithm, comparable to other more complex techniques like neural networks [35]. This classifier has an additive smoothing parameter, called alpha, which value was set to 0.5.
- Logistic Regression (LR). Models the probability of events’ occurrence as a linear function of a set of predictor variables and can be used to predict the value of dependent variables. Because this algorithm builds a prediction model instead of a estimated point of dependent variables, it is used as a effective classifier [36]. Parameter *C* corresponding to the inverse of regularization strength was set to 1.0.
- Support Vector Machine (SVM). This algorithm uses a nonlinear matching method to transform the original dataset into a higher dimension—namely, a hyper-plane that acts as the decision boundary for partitioning data into classes [37]. Support vector machines are used to determine an optimal decision boundary to partition data into different classes. It is important to mention that SVM does not generate class probabilities, but they were calculated while using the algorithm proposed by [38]. Radial base was used as kernel, with kernel coefficient (gamma) set to 0.00001 and the penalty parameter of the error term (*C*) set to 3500.

### 3.4. Ensemble Classification

Ensemble classification considers the output of several classifiers, whose individual decisions are combined in some way—typically by weighted or un-weighted voting—in order to classify new examples [12]. In this work, ensemble classification is used to classify new tweets, consisting in n=3 classifiers denoted by $C_{1},C_{2},\ldots ,C_{n}$. For each tweet ${\hat{t}}_{q},q=1,2,\ldots ,t$ classifier $C_{i},i=1,2,\ldots ,n$ generates *m* probabilities $P_{i,j},j=1,2,\ldots ,m$. $P_{i,1}$ indicates the probability generated by the classifier $C_{i}$ that the *q*-th tweet ${\hat{t}}_{q}$ belongs to class $L_{1}$, $P_{i,2}$ the probability that it belongs to the class $L_{2}$ and so on for the *m* classes, as shown in Table 2.

The proposed weighting is a soft weighting scheme, in such a way that it weights the *m* probabilities generated by classifier $C_{i},i=1,2,\ldots ,n$ with weights $w_{i},i=1,2,\ldots ,n$. See Table 3.

### 3.5. Accuracy of the Ensemble Classifier

There are different metrics to evaluate an automatic learning model: accuracy, model error, completeness, precision, recall, and F1 measure are some of them. In this work, accuracy will be used to evaluate the ensemble classifier.

Once the probabilities generated by the *n* classifiers are weighted, the probability weighted by class can be obtained for the *q*-th tweet that is described in (1).

(1) $${\hat{P}}_{j}=\sum_{i=1}^{n}P_{i,j}w_{i},\;j=1,\ldots ,m$$

The prediction of the ensemble learning for the *q*-th tweet is the maximum probability weighted by class described in (2).

(2) $$D_{q}=max\left\{{\hat{P}}_{j}\right\},\;j=1,\ldots ,m$$

Given the set of predictions $D=\{D_{1},D_{2},\ldots ,D_{t}\}$ of the ensemble learning and the set of real classes $R=\{R_{1},R_{2},\ldots ,R_{t}\}$ of tweets ${\hat{t}}_{q}$, q=1,2,…,t, it is possible to know the number of intersections between these two sets, as described in (3).

(3) e=|D∩R|

Finally, the accuracy of the ensemble learning with weights $\vec{w}$ is described in (4).

(4) $$J\left(\bar{w}\right)=\frac{e}{t}$$

### 3.6. Maximum Theoretical Accuracy

The intention of this work is to maximize the accuracy of the ensemble classifier. However, it is good to have a reference to know how far it is possible to maximize this accuracy. For this, the maximum accuracy of *n* classifiers was calculated. The prediction of classifier *i* for the *q*-th tweet is described in (5).

(5) $$a_{i,q}=max\left\{P_{i,j}\right\}$$

The predictions of *n* classifiers for the *q*-th tweet can be calculated, as shown in (6).

(6) $$A_{q}=\left\{a_{1,q},a_{2,q},\ldots ,a_{n,q}\right\}$$

If the cardinality of the intersection of the set $A_{q}$ with the real class $R_{q}$ of the *q*-th tweet is greater than zero, it is considered that there is a coincidence between any of the predictions of the *n* classifiers with the real class of the q-th tweet, as described in (7).

(7) $$d_{q}=\left\{\begin{array}{cc} 0, & \mathrm{if}\;|A_{q}\cap R_{q}|=0\\ 1, & \mathrm{if}\;|A_{q}\cap R_{q}|>0 \end{array}\right.$$

The maximum theoretical accuracy of *m* classifiers is described in (8).

(8) $$TA=\frac{\sum_{q=1}^{t}d_{q}}{t}$$

As an example, we calculate the maximum accuracy of three classifiers for five tweets ${\hat{t}}_{q}$, with real classes $R_{1},R_{2},R_{3},R_{4},R_{5}$, and predictions from three classifiers $a_{i,k}$ with cardinalities $d_{q}$, as shown in Table 4. For this example, the maximum accuracy described in (9) is obtained.

(9) $$\frac{3}{5}=0.6$$

## 4. Evolutionary Optimization of the Weighted Ensemble Classification

A mono-objective optimization problem is considered in order to maximize the accuracy of the ensemble classifier. This can be described in a general way as maximizing $J(\vec{w})$ subject to (10) and (11).

The design goal $J(\vec{w})$ considers the *e* matches between the set of predictions $D=\{D_{1},D_{2},\ldots ,D_{t}\}$ of the ensemble learning, and the set of real classes $R=\{R_{1},R_{2},\ldots ,R_{t}\}$ of the test tweets $t_{q},\;q=1,2,\ldots ,t$, where $\vec{w}$ are the weights that must be adjusted to maximize *J*, as defined in (4).

The design variables are the weights that are assigned to classifiers $C_{i},\;i=1,\ldots ,n$. The set of design variables are grouped in vector $\vec{w}=\left[w_{1},w_{2},\ldots w_{n}\right]$.

It is necessary to narrow the search space, establishing boundaries for the design variables, in order to find optimal solutions to real-world problems. In the case of this problem, these limits are established as the inequality constraints (10).

(10) $$g_{i}:0<w_{i}<1,\;\forall i=1,\ldots ,n$$

Another restriction that must be met is that the sum of the weights $\vec{w}$ assigned to classifiers $C_{i},i=1,\ldots ,n$ must be equal to 1. This constraint is described in (11).

(11) $$h_{1}:\sum_{i=1}^{n}w_{i}=1$$

### 4.1. Differential Evolution Algorithm

Differential Evolution (DE) is an evolutionary algorithm proposed by Rainer Storn and Kenneth Price to solve global optimization problems in continuous search spaces [39]. DE has characteristics of robustness, precision, and speed of convergence that have made it attractive, not only to solve problems with continuous search spaces [40,41], but also discrete spaces [42,43]. DE begins with a set of solutions, called parents population, to which processes of crossing, mutation and selection are applied to create child populations that approach optimum solutions in an iterative process. The parameters of DE are: population size *NP*, maximum number of generations $G_{\mathrm{Max}}$, number of crossings $C_{r}$, and a factor of scale *F*.

There are different variants of DE, being the most popular rand/1/bin—the one used in this work. The word rand indicates that the three individuals selected to calculate the mutation value are selected randomly, 1 the number of pairs of solutions chosen, and bin that a binomial recombination is used [44].

In this work, DE creates an initial matrix population $W_{G}=\left[{\vec{w}}_{G}^{1},\ldots ,{\vec{w}}_{G}^{NP}\right]\in I\;R^{NP\times m}$ with *NP* individuals, called population of parents. Each individual of $W_{G}$ contains the design variables $\vec{w}$ generated randomly, as described in (12) and (13), respecting the inequality constraints (10) and the equality constraint (11). In the mutation process a mutant individual ${\vec{v}}_{G}^{i}$ is created with three parent individuals (${\vec{w}}_{G}^{a}$, ${\vec{w}}_{G}^{b}$ and ${\vec{w}}_{G}^{c}$) different to the current father ${\vec{w}}_{G}^{i}$ and the scale factor *F*. In the crossing process, the crossing factor $C_{r}$ is considered to determine whether the gene inherited from the individual child ${\vec{u}}_{G}^{i,j}$ is taken from the mutant individual ${\vec{v}}_{G}^{i,j}$ or from the parent individual ${\vec{w}}_{G}^{i,j}$. Subsequently, it is verified if the child individual ${\vec{u}}_{G}^{i}$ complies with constraints (10) and (11). If not, ${\vec{u}}_{G}^{i}$ is randomly generated with (12) and (13). Finally, in the selection process, the individual parent of the next generation ${\vec{w}}_{G+1}^{i}$ will be the individual with greater accuracy comparing the child individual ${\vec{u}}_{G}^{i}$ and the parent individual ${\vec{w}}_{G}^{i}$. These processes continue iteratively while $G<=G_{\mathrm{Max}}$. The population of the maximum generation $W_{G_{\mathrm{Max}}}$ has the individuals with better accuracy for *Max* generations. Algorithm 1 shows the complete pseudo-code of the implementation of the DE/rand/1/bin algorithm in order to optimize the weights of the ensemble classifier.

(12) $$y_{i}=rand\left(0,10\right)\;\forall i=1,\ldots ,n$$

(13) $$w_{i}=\frac{y_{i}}{\sum_{i=1}^{n}y_{i}}\;\forall i=1,\ldots ,n$$

**Algorithm 1:** Pseudocode of the DE algorithm for the evolutionary optimization of the ensemble classifier.
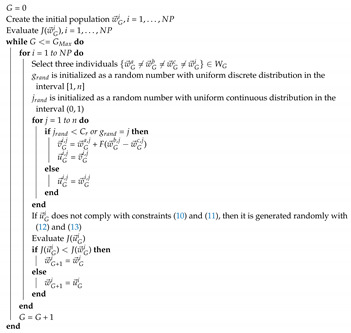


### 4.2. K-Fold Cross-Validation and Stratified K-Fold Cross-Validation

It is important to estimate the performance of classifiers in order to select the most appropriate scheme. A common strategy for this purpose is to use *k*-fold cross-validation, in which a dataset *S* is split in *k* mutually exclusive subsets, called folds, $S_{1}$, $S_{2}$, …, $S_{k}$ of approximately the same size [45]. Subsequently, classifiers are trained and tested *k* times; each time g∈{1,2,…,k}, it is trained on the training subset $S-S_{g}$, and tested on $S_{g}$ (testing subset).

A variation of this strategy, called stratified *k*-fold cross-validation, considers the distribution of classes to create the folds [45]. The folds in this strategy are evenly distributed, so that they contain approximately the same proportions of labels as the original dataset. In our proposal, for both strategies *k* is equal to 10, which is, the training set is divided in 10 folds.

Both of the strategies show the robustness of classifiers and the average accuracy of folds is a good estimator of expected performance on the test set. Therefore, we apply the evolutionary optimization method described in Algorithm 1 on each fold to calculate the best weighting scheme. Selection of the best weighting scheme is described in the following subsection.

### 4.3. Best Weights Selection Strategy

Evolutionary optimization algorithms provide a set of good solutions. From these solutions, the one that maximizes (or minimizes, depending on the problem) the objective function must be selected. A simple solution could just select the weighting scheme that maximizes accuracy, but this weighting scheme would have been calculated on a single fold of a test set, and there is no certainty that it could obtain the same good results in the test subsets from other folds. To avoid this bias in selecting the best solution, the next next steps are followed:

1. Train the classifiers described in Section 3.3 with each of the 10 training sets.
2. Use Algorithm 1 to determine the weighting schemes that maximizes accuracy on each of the 10 testing sets. In this step 10 candidates for best weighting scheme are obtained, one for each testing fold.
3. Use the obtained weighting schemes of each test set on the ensemble to classify the tweets of remaining nine test sets.
4. Calculate the average accuracy obtained by each weighting scheme of the ensemble on the test sets.
5. Select the weighting scheme with the best average accuracy.

As well as cross-validation ensures the robustness of the classifiers, we consider that the selection strategy described above takes advantage of the diversity of samples on the folds to provide a global solution (The apparently straightforward selection strategy of averaging weights from the best weighting vectors in each fold was also tested, with no satisfactory results.).

The complexity of Algorithm 1 is calculated as $O(G_{Max}\cdot NP\cdot n)$, where $G_{Max}$ is the number of generations for crossover and mutation of individuals of the NP population, and *n* is the number of design variables that corresponds to the number of weights to be assigned to the classifiers (in this case, 3).

## 5. Experiments And Results

Our goal is to be able to correctly classify the polarity of tweets of the test set *Z* of the TASS corpus, as described in Section 3.1. In order to do so, first the classifiers are trained and adjusted on the training corpus *E*. Experiments and results on this set are described in Section 5.1; afterwards, the experiments on the test set *Z* are described in Section 5.2.

### 5.1. Experiments on the E Set

Several strategies can be explored for training and adjusting the ensemble learning scheme with differential evolution weight selection. The number of individuals and generations can be changed (See Section 5.1.1), as well as the way of creating folds (see Section 5.1.2). With these experiments, the optimal weighting scheme $\vec{w}$ is sought. Subsequently, it will be applied to classify the TASS test set *Z*, as described in Section 5.2.

#### 5.1.1. Random Folds

Table 5 shows the results of the first experiment with the training set of the TASS corpus *E* and 10 folds. From rows one to three, the accuracy obtained by the NB, LR, and SVM classifiers is observed independently. The fourth row (*Hard w*) shows the accuracy obtained by ensemble classification with the same classifiers using a hard weighting (same weights for all classifiers). Row 5 (*Soft w:20–300*) shows the accuracy obtained by the ensemble classification using the weighting scheme of the best individual after the process of evolutionary optimization. For this selection a random initial population of 20 individuals over 300 generations was used and the experiment was run 30 times in order to ensure robustness in the results. The total execution time was about 16 h in a 20 core dual-processor Intel Xeon E5-2690V2 Server (TEN CORE @ 3.0 GHz). The average of these 30 runs is reported. For results shown in Row 6 (*Soft w:200–1000*) the population was increased from 20 individuals to 200, and generations were increased from 300 to 1000 with the intention of achieving greater diversity in the population. As well as in Row 5 (*Soft w:20–300*), the experiment was run 30 times. The execution time was similar to the previous experiment. Because these changes did not have a significant effect in results, no more increases in generations or individuals were tested. Row 7 (*TA*) shows the maximum theoretical accuracy that was obtained by ideally selecting the correct result, if provided by any classifier (see Section 3.6). The last column shows the average accuracy of the classifiers and the ensemble learning. The highest accuracy for each independent classifier on each fold is highlighted in italics, while the best overall accuracy (without considering TA) is shown in bold.

#### 5.1.2. Stratified Folds

**Figure 1:**
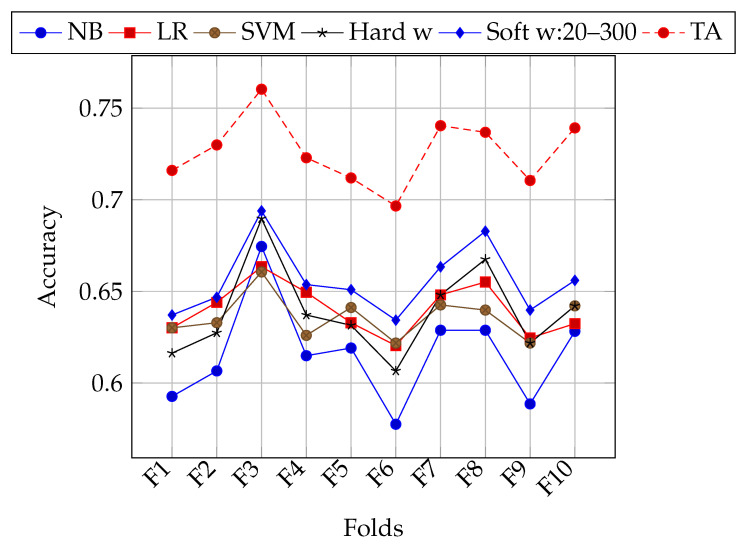
Accuracy of each classifier, different weighting schemes, and maximum theoretical accuracy on each fold of the *E* set of the TASS corpus.

**Figure 2:**
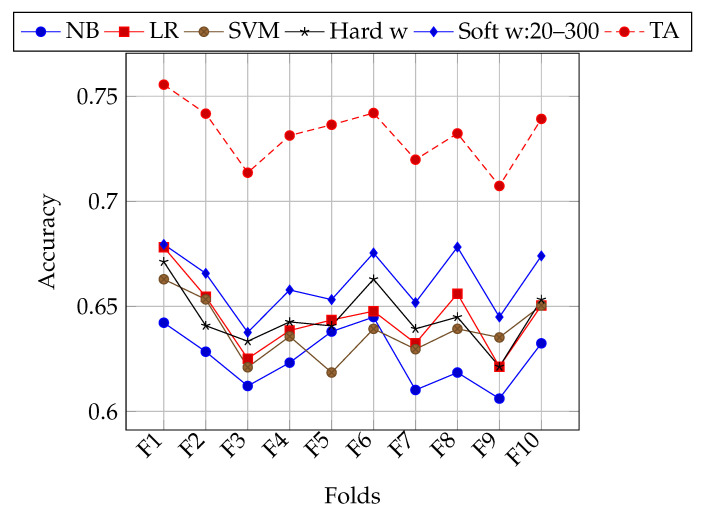
Accuracy of each classifier, different weighting schemes, and maximum theoretical accuracy on each stratified fold of the *E* set of the TASS corpus.

The folding strategy in previous experiments consisted of randomly selecting tweets from the *E* set of the TASS corpus. As can be seen in Figure 1, for some folds all classifiers in general achieve better accuracy. This might be due to the class bias of tweet polarities (unbalanced number of classes). Experiments with stratified k-folding were performed in order to lessen the impact of this bias on classification accuracy. In Table 6, results of using stratified k-folding are shown. Figure 2 shows performance for each fold using stratification. Both of the configurations of 20 individuals and 300 generations, and 200 individuals and 1000 generations were used. In general, soft weighting improves the classification accuracy on stratified folds as well. Nevertheless, there is still an heterogeneous performance for different folds.

**Figure 3:**
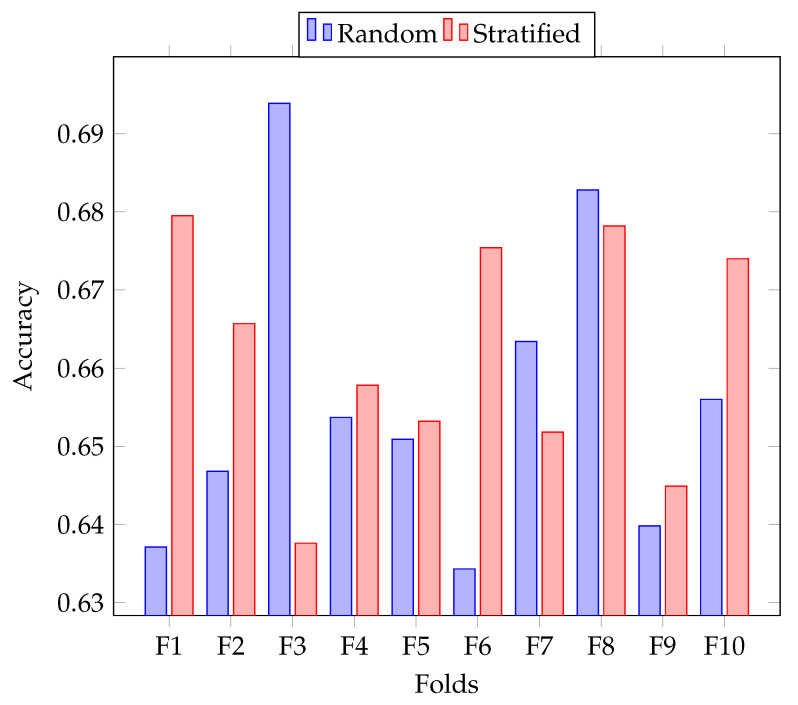
Accuracy of *Soft w:20–300* on both random and stratified folding strategy for each created fold of the *E* set of the TASS corpus.

Figure 3 shows a comparison of accuracy obtained by *Soft w:20–300* on both folding strategies, random and stratified. Stratified folding improved the performance on most of the folds, but decreased on others. On average, the accuracy on random folds was 0.6558, while average accuracy on stratified folds was 0.6618.

For each fold, different soft weights were found. In the next section it is explained how the best weight on each folding strategy is selected in order to classify the tweets of the final test on the *Z* set.

### 5.2. Experiments on the *Z* Set

For each experiment described in the previous sections, a vector of optimal weights $\vec{w}$ was obtained for each fold. The strategy detailed in Section 4.3 was applied for each experiment in order to select the best set of weights. The selected weighting vector on the random folds (from the *soft w:20–300* experiment) was $\vec{w_{r}}$ = [0.1713, 0.0380, 0.7905], while the vector corresponding to the stratified folds (using 20 individuals and 300 generations) was $\vec{w_{s}}$ = [0.1345, 0.0340, 0.8313]. Each value in this vector corresponds to the weight of each classifier, namely NB, LR, and SVM.

It can be seen that, in both $\vec{w_{r}}$ and $\vec{w_{s}}$, SVM is given a predominant weight (0.7905 and 0.8313 respectively); this is interesting, because this classifier obtained better average accuracy than NB, but lower than LR.

Once the weights were determined, tweets in the test set were classified with each classifier, and they were then assembled in a voting scheme with $\vec{w_{r}}$ and $\vec{w_{s}}$ weights, respectively. The results are shown in Table 7. As can be seen $\vec{w_{s}}$ based on stratified folds (which obtained better results in the training set *E*) also yielded the best result in the test set *Z*.

### 5.3. Comparison with Other Works

Table 8 presents the best results reported by other systems on the same task. To our knowledge, the best accuracy reported so far is 0.726 by the LIF system. However, in order to fairly compare these systems, it is necessary to consider the external resources they are using to improve classification. For example, the LIF system uses external affective lexicons, such as ElhPolar [46], SSL [47], LYSA [48], MPQA [49], and HGI [50]. A similar situation occurs with the first four systems with the highest accuracies. Isolation from the effect of other resources is desirable, as, in principle, we aim to improve classification accuracy by adjusting weights of a classification ensemble. In that sense, we are comparable to the LYS, SINAI-DW2Vec, and INGEOTEC systems. Our proposed classification method with soft weights on stratified k-folds overcomes the accuracy of these systems.

Additionally, Table 9 gives a brief description of the tools used by the best methods for classifying polarity tweets on the TASS task. The first column after accuracy shows the maximum number of n-gram features being used. In our work, we used only bag of words, which is equivalent to using unigrams. We are not using a Named-Entity-Recognizer module or NLP techniques (such as lemmatization, using parts-of-speech tags, etc.). We do not handle negation with any particular method. Other works use feature augmenting methods that are based on deep learning (Word2Vec [55], Doc2Vec [56], GloVe [57]), distributional methods (LDA [58], LSI [59]), or other feature weighing methods (TF·IDF [60]). In this work, none of these was used.

The last column of Table 9 shows a very compact survey of the classifiers used by each system. Most works use Support Vector Machines (SVM). The first system (LIF) uses a ensemble of SVM, and Convolutional networks with skipgrams, bag of words, and vectors obtained from GloVe [57]. These results are fed to an SVM classifier. ELiRF, the second best system combines the output of several SVM classifiers with different parameters, and then this information is classified in cascade with another SVM classifier.

### 5.4. Discussion

The Differential Evolution strategy for optimizing the weights in a soft-voting ensemble was able to overcome performance of the individual classifiers. As expected, in the *E* set performance was better for the soft voting scheme, compared with hard weighting. Specifically, this latter achieves 66.57% accuracy, while the best weights obtained by Differential Evolution reach 67.71%.

Additionally, two different ways of partitioning information for finding the best weights were explored. One was based on random k-folds, and other on stratified k-folding. Stratified k-folds tend to improve the final classification. The latter strategy had better performance on the *E* set (66.19% vs. 65.61%), and the weight vector *Soft ${\vec{w}}_{s}$* calculated on these folds slightly contributed to obtain a better classification on the *Z* corpus (67.71% vs. 67.68%). In both folding strategies, the soft weighting always outperformed the hard weighting scheme.

We experimented with the InterTASS corpus of 2018 (Spanish) in order to test our solution with a different corpus [61]. We applied the DE:Soft ${\vec{w}}_{s}$ method without recalculating weights. The results are shown in Table 10. From this table, it can be seen that despite the full process of adjusting weights was not carried out, our method outperformed some of the neural-network-based methods (retuyt-cnn).

We have calculated the statistical significance of our experiments while using the STAC platform [62] considering the different results we obtained separately with each classifier, hard weights, and soft weights. With the Shapiro–Wilk test [63], we obtained that the null hypothesis is rejected with a level of significance of 0.093, while for the Kolmogorov–Smirnov test [64], it is rejected with ρ<0.001.

## 6. Conclusions

In this paper, we presented a method to optimize weights for a classification ensemble. When compared with other methods, DE:Soft ${\vec{w}}_{s}$ is able to obtain state of the art accuracy, given that no external resources are being used. As a future work, it would be interesting to assess the effect of using our proposed method along with external resources in order to further improve scores for this task.

In general, this proposal could be used for problems where training data are relatively small when compared with the amounts required for other state of the art methods, such as deep learning. Automatic optimization of weights for different classifiers allows for easily adapting this method for other problems, including those with multiclass labels.

In both ${\vec{w}}_{r}$ and ${\vec{w}}_{s}$, SVM is notoriously given a predominant weight, although it is interesting to see that this is not the best overall classifier if used alone. In this case, LR would be a better choice (see Table 5 and Table 6). Additionally, one of the best reported systems (GTI-GRAD) uses LR as its main classifier. This suggests a deeper by-case analysis that may enable classifiers to specialize in particular cases, along with a meta-classifier that dynamically adjusts weights for each case. Another option is to create separate classifiers per class; this is left as future work. Other improvements to the Differential Evolution algorithm, such as different ways of partitioning data, are also considered for further exploration.

## Figures and Tables

**Table 1:** Opinion polarity distribution of tweets in TASS corpus.

Polarity	*E*		*Z*	
	Frecuency	Tweets	Frecuency	Tweets
Positive	39.94%	2884	36.57%	22,233
Negative	30.22%	2182	26.06%	15,844
None	20.54%	1483	35.22%	21,416
Neutral	9.28%	670	2.15%	1305

**Table 2:** Probabilities generated by the classifier $C_{i}$, of which the *q*-th tweet ${\hat{t}}_{q}$ belongs to each of the $L_{j},j=1,2,\ldots ,m$ classes.

	$L_{1}$	$L_{2}$	⋯	$L_{m}$
$C_{i}$	$P_{i,1}$	$P_{i,2}$	⋯	$P_{i,m}$

**Table 3:** Weighting scheme of ensemble classification.

	$L_{1}$	$L_{2}$	⋯	$L_{m}$
$C_{1}$	$P_{1,1}w_{1}$	$P_{1,2}w_{1}$	⋯	$P_{1,m}w_{1}$
$C_{2}$	$P_{2,1}w_{2}$	$P_{2,2}w_{2}$	⋯	$P_{2,m}w_{2}$
⋯	⋯	⋯	⋯	⋯
$C_{n}$	$P_{n,1}w_{n}$	$P_{n,2}w_{n}$	⋯	$P_{n,m}w_{n}$

**Table 4:** Predictions of three classifiers for five tweets.

${\hat{t}}_{1}$	${\hat{t}}_{2}$	${\hat{t}}_{3}$	${\hat{t}}_{4}$	${\hat{t}}_{5}$
$R_{1}=NONE$	$R_{2}=NEU$	$R_{3}=P$	$R_{4}=P$	$R_{5}=N$
$a_{1,1}=P$	$a_{1,2}=NONE$	$a_{1,3}=P$	$a_{1,4}=N$	$a_{1,5}=N$
$a_{2,1}=N$	$a_{2,2}=P$	$a_{2,3}=P$	$a_{2,4}=NONE$	$a_{2,5}=N$
$a_{3,1}=NONE$	$a_{3,2}=P$	$a_{3,3}=N$	$a_{3,4}=NEU$	$a_{3,5}=N$
$d_{1}=1$	$d_{2}=0$	$d_{3}=1$	$d_{4}=0$	$d_{5}=1$

**Table 5:** Results on random folds of the TASS training set *E*.

	F1	F2	F3	F4	F5	F6	F7	F8	F9	F10	Average
NB	0.5927	0.6066	*0.6745*	0.6149	0.6191	0.5775	0.6288	0.6288	0.5886	0.6282	0.6159
LR	*0.6301*	*0.6440*	0.6634	*0.6495*	0.6329	0.6204	*0.6481*	*0.6551*	*0.6246*	0.6324	0.6400
SVM	*0.6301*	0.6329	0.6606	0.6260	*0.6412*	*0.6218*	0.6426	0.6398	0.6218	*0.6421*	0.6358
Hard w	0.6163	0.6274	0.6897	0.6371	0.6315	0.6066	0.6481	0.6675	0.6218	0.6421	0.6388
*Soft w:20–300*	**0.6371**	**0.6468**	0.6939	**0.6537**	**0.6509**	**0.6343**	**0.6634**	**0.6828**	**0.6398**	**0.6560**	**0.6558**
*Soft w:200–1000*	**0.6371**	**0.6468**	**0.6966**	**0.6537**	**0.6509**	**0.6343**	**0.6634**	**0.6828**	**0.6398**	**0.6560**	**0.6561**
TA	0.7160	0.7299	0.7603	0.7229	0.7119	0.6966	0.7409	0.7368	0.7105	0.7392	0.7265

**Table 6:** Results on stratified folds of the training set of the TASS corpus *E*.

	F1	F2	F3	F4	F5	F6	F7	F8	F9	F10	Average
NB	0.6422	0.6284	0.6127	0.6232	0.6380	0.6449	0.6102	0.6185	0.6061	0.6324	0.6256
LR	*0.6781*	*0.6546*	*0.6251*	*0.6385*	*0.6435*	*0.6477*	*0.6324*	*0.6560*	0.6213	*0.6504*	0.6447
SVM	0.6629	0.6533	0.6210	0.6357	0.6185	0.6393	0.6296	0.6393	*0.6352*	*0.6504*	0.6385
Hard w	0.6712	0.6408	0.6334	0.6426	0.6407	0.6629	0.6393	0.6449	0.6213	0.6532	0.6450
*Soft w:20–300*	**0.6795**	**0.6657**	**0.6376**	**0.6578**	**0.6532**	**0.6754**	**0.6518**	**0.6782**	**0.6449**	0.6740	**0.6618**
*Soft w:200–1000*	**0.6795**	**0.6657**	**0.6376**	**0.6578**	**0.6532**	**0.6754**	**0.6518**	**0.6782**	**0.6449**	**0.6754**	**0.6619**
TA	0.7555	0.7417	0.7136	0.7313	0.7364	0.7420	0.7198	0.7323	0.7073	0.7392	0.7319

**Table 7:** Results of the experiment with *Z* set of the TASS corpus. **Soft** ${\vec{w}}_{r}$ shows results with soft weights calculated from random folds while those of **Soft** ${\vec{w}}_{s}$ were calculated from stratified folds.

Method	Accuracy
**NB**	0.6384
**LR**	0.6712
**SVM**	0.6721
**Hard** $\vec{w}$	0.6657
**Soft** ${\vec{w}}_{r}$	0.6768
**Soft** ${\vec{w}}_{s}$	**0.6771**

**Table 8:** External resources used and Accuracy for TASS Task 1, 4 classes, *Z* corpus. Best run reported for each system.

System	Accuracy	ElhPolar [46]	SOCAL [1]	iSOL [51]	SSL [47]	Own	DAL [52]	LYSA [48]	ML Senticon [53]	Semeval 2013 [54]	MPQA [49]	HGI [50]
LIF	0.726	✓			✓			✓			✓	✓
ELiRF	0.725				✓				✓	✓		
GTI-GRAD	0.695		✓	✓		✓	✓					
GSI (aspect)	0.691	✓	✓	✓	✓				✓			
**DE:Soft** ${\vec{w}}_{s}$ **(us)**	0.677											
LYS	0.664											
DLSI	0.655					✓						
SINAI-DW2Vec	0.619											
INGEOTEC	0.613											

**Table 9:** Classifiers used for systems in Table 8. (LR = Logistic Regression, SVM = Support Vector Machines, ME = MaxEnt, SG = SkipGrams).

System	Accuracy	max n-gram	NER	NLP	Negation	Word2Vec	Doc2Vec	GloVe	TF·IDF	LDA	LSI	Classifier
LIF	0.726	1		✓		✓		✓			✓	(SVM SG Cbow)→SVM
ELiRF	0.725	1		✓					✓			SVM (+ SVM)
GTI-GRAD	0.695	2		✓								LR
GSI (aspect)	0.691	1	✓	✓	✓							SVM
**DE:Soft** ${\vec{w}}_{s}$ **(us)**	0.677	1										DE: (NB, LR, SVM)
LYS	0.664	1		✓								Logistic regression L2-LG
DLSI	0.655	2		✓								SVM
SINAI-DW2Vec	0.619	1				✓	✓					SVM
INGEOTEC	0.613	5							✓	✓	✓	SVM

**Table 10:** Results of our proposed method with the InterTASS ES corpus, as compared with top results [61].

System	M. F1	Acc.	System	M. F1	Acc.
elirf-es-run-1	0.503	0.612	atalaya-lr-50-2-roc	0.455	0.595
retuyt-lstm-es-1	0.499	0.549	ingeotec-run1	0.445	0.530
retuyt-combined-es	0.491	0.602	atalaya-svm-50-2	0.431	0.583
atalaya-ubav3-100-3-syn	0.476	0.544	itainnova-cl-base	0.383	0.433
DE:Soft ${\vec{w}}_{s}$	0.461	0.585	itainnova-cl-proc1	0.320	0.395
retuyt-cnn-es-1	0.458	0.592			
